# Spatially organized lymphocytic microenvironments in high grade primary prostate tumors

**DOI:** 10.1101/2025.09.21.677465

**Published:** 2025-09-21

**Authors:** Ali Amiryousefi, Jeremiah Wala, Jia-Ren Lin, Brian William Labadie, Aishwarya Atmakuri, Zoltan Maliga, Eamon Toye, Kiranj Chaudagar, Madeleine S. Torcasso, Shannon Coy, Giuseppe Nicolo Fanelli, Brigette Kobs, Fabio Socciarelli, Andreanne Gagne, Eliezer M. Van Allen, Akash Patnaik, Peter Sorger

**Affiliations:** 1 Laboratory of Systems Pharmacology, Department of Systems Biology, Harvard Medical School, Boston, MA 02115, USA; 2 Ludwig Center at Harvard, Harvard Medical School, Boston, MA 02115, USA; 3 Dana Farber Cancer Institute, 450 Brookline Ave, Boston, MA 02215, USA; 4 University of Chicago, 5841 S Maryland Ave, Chicago, IL 60637, USA; 5 Department of Pathology, Brigham and Women’s Hospital, Harvard Medical School, Boston, MA 02115, USA; 6 Department of Pathology and Laboratory Medicine, Weill Cornell Medicine, New York, NY, 10021, USA; 7 Division of Pathology, Department of Translational Research and New Technologies in Medicine and Surgery, University of Pisa, Pisa, 56126, Italy; 8 University Institute of Cardiology and Pulmonology of Quebec (CRIUCPQ), Laval University, 2725 Chemin Sainte-Foy, Quebec City, QC G1V 4G5, Canada

**Keywords:** Prostate cancer, tumor microenvironment, multiplex imaging, immune infiltration, spatial biology

## Abstract

The spatial organization and composition of the tumor-immune microenvironment (TME) play a critical role in shaping the progression of many solid cancers, but the organization of the TME in primary prostate cancer (PCa) remains poorly characterized. We therefore profiled the abundance and spatial distributions of major cell types involved in adaptive immunity in 29 radical prostatectomy specimens stratified into high (HGG; n=14) and low Gleason-grade (LGG; n=15). Compared to LGG, HGG PCa exhibited significantly greater B and T cell infiltration with many immune cells organized into clusters, some of which resembled tertiary lymphoid structures (TLSs). In HGG tumors, these clusters were dense, symmetric, rich in PD-1^+^ T cells, and frequently proximate to the tumor compartment. LGG clusters were less well organized, and T cell depleted. Thus, a subset of high-grade PCa harbor organized immune clusters that may play a role in tumor control and contain therapeutically targetable T and B cells.

## INTRODUCTION

Prostate cancer (PCa) is often considered to be an immunologically “cold” tumor with no established role for immunotherapy except in the rare case of microsatellite instability-high (MSI-H) tumors^1^. However, expert pathology review of hematoxylin and eosin (H&E) stained tissue sections has long recognized the presence of tumor-infiltrating immune cells in primary untreated prostate cancer (PCa) obtained from surgical resections^2–4^. Conventional IHC studies and more recently multiplexed IHC and multiplexed immunofluorescence (IF) have further confirmed the presence of tumor infiltrating lymphocytes^5–15^, immunosuppressive regulatory T cells^16–18^ and tumor-associated macrophages^19–21^, among other cell types. Despite the presence of immune cells in the tumor microenvironment (TME) of PCa, immune checkpoint inhibition (ICI) trials have been underwhelming in this setting^22,23^ and there is no established role for tumor immune profiling in guiding treatment of PCa: pathologic diagnosis remains focused on tumor morphology using H&E images and tumor-specific markers (e.g. AMACR, NKX3).

At the same time, tumor-infiltrating CD4^+^ and CD8^+^ T cells in primary PCa have been shown to express the immune checkpoint marker and ICI target PD-1^24^. A minority of primary PCa tumors are associated with increased expression of the PD-1 ligand (PD-L1) and also harbor CD8^+^ immune cells^13,25^, suggesting that the targets of PD-1 or PD-L1 directed ICIs can be present in primary PCa. However, bulk RNA-seq studies have shown that prostate tumors carry lower T cell fractions and higher macrophage and neutrophil signatures than adjacent benign tissue^26^. Moreover, single-cell RNA-seq (scRNA-seq) surveys of primary PCa have suggested the presence of an immune-suppressive microenvironment rich in macrophages and inflammatory monocytes with most CD8^+^ T in an exhausted state^27,28^. Thus, extent to which PCa contains potentially ICI-targetable T cells remains unclear.

The TME of solid tumors is characterized not only by cellular composition but also by spatial organization. The spatial architecture of prostate cancer is assessed by Gleason grade, which describes tumor growth patterns ranging from well-formed separated glands (Gleason 3) to a disorganized pattern of tumor cells as individual cells, cords, or solid sheets without gland formation (Gleason 5)^29^. Gleason grade is the key diagnostic feature for risk-stratification and clinical decision making in treatment-naïve prostate cancer. In some other solid cancers, the spatial properties of the immune system can be prognostic (and in some cases predictive of response to immune checkpoint inhibitors; ICIs)^30–33^, most notably in the presence of tertiary lymphoid structures (TLS)^34^ in melanoma and colorectal cancer (CRC)^35^, and tumor-CD8^+^ T cell proximity in non-small cell lung cancer^36^. Some PCas have been shown to contain immune clusters ranging from small immune aggregates^25^ to TLS-like structures^37^. For example, in a cohort of primary PCa selected for high PD-L1 expression, Calagua et al. identified niches of MHC-II^+^ dendritic cells in spatial proximity to PD-1^+^ TCF1^+^ stem-like CD8^+^ T cells^25^. These niches were associated with increased tumor-infiltrating lymphocytes (TILs), which is consistent with the immune cell clusters in PCa representing sites of antigen cross-presentation and tumor reactive T cell responses. Larger immune clusters have also been identified in both low- and high-grade PCa with multiplex IF, and at least a subset of these structures display canonical TLS features including proliferating CD20^+^ B-cell follicles and follicular dendritic cells^37^. These TLS-like structures are also identifiable in H&E images, with associated gene signatures supporting the identification of these primarily as T and B cell clusters^4^. Moreover, recent multiplexed IF imaging of 13 primary PCa tumors identified cellular neighborhoods comprised of mast cells, regulatory T cells and M2-like macrophages, suggesting a spatial organization to immunosuppression programs previously identified by RNA-seq^38^.

A limitation of studies to date is that criteria for identifying statistically significant aggregates of immune cells and patterns of T and B cell interaction have not been well defined– particularly in PCa. To address this, we combined highly multiplexed cyclic immunofluorescence (CyCIF)^39^ profiling with a set of intuitive spatial analysis methods designed to precisely characterize the absolute levels and relative distributions of immune cells in the TME; these methods included K nearest neighbor (KNN) classification of dense immune clusters, DBSCAN (Density-Based Spatial Clustering of Applications with Noise) of looser associations, and the centered L-function (CLF) to precisely distinguish organized and random distributions. We examined 29 radical prostatectomy specimens enriched for high Gleason grade (HGG; Gleason grade ≥ 4+4) tumors; these HGG tumors represent ~ 20% of primary PCa tumors but half of PCa mortality^40,41^. We found that HGG PCa was significantly more likely than low Gleason Grade (LGG; Gleason grade ≤ 4+3) PCa to contain immune cell clusters (ICs) with three key features of a spatially organized antitumor immune responses: (i) dense TILs comprising B and T cells (in different proportions); (ii) proliferating B cells and progenitor exhausted T cells (T_PEX_) within the cores of these infiltrates; and (iii) granzyme-B positive (GZB^+^) cytotoxic T cells in contact with tumor cells. Relative to mismatch repair deficient (dMMR) colorectal cancers, which are an archetype of an ICI-responsive tumor rich in TLSs, we found that HGG PCa was similarly infiltrated by B cells but had fewer CD4^+^ and CD8^+^ T cells. We conclude that a subset of high-risk primary PCa is immune-rich with features associated with active immune surveillance including T cell infiltration and TLS-like clusters, raising the question of whether these tumors may harbor a therapeutically targetable immune program not yet unlocked by existing ICI strategies.

## RESULTS

### Spatial immunophenotyping PCa reveals high lymphocytic infiltration in a subset of high-grade tumors

**Fig. 1 |:**
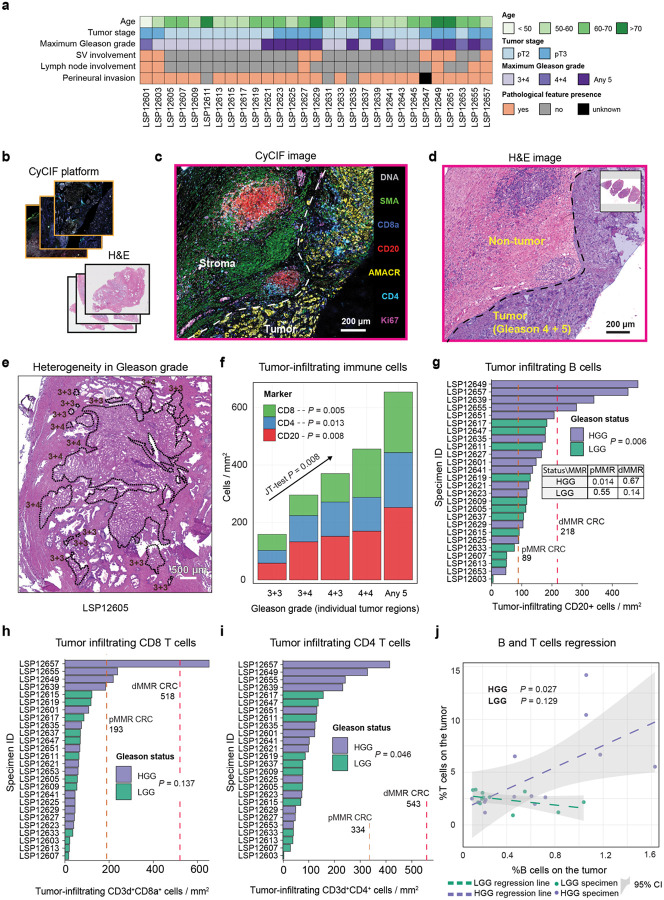
Whole slide t-CyCIF imaging reveals elevated B and T cell infiltration in high Gleason grade (HGG) PCa. **a** Cohort overview with key clinical annotations. **b** Experimental workflow: whole-slide cyclic multiplex immunofluorescence (CyCIF) and adjacent H&E imaging was performed on whole-slide radical-prostatectomy specimens. **c** CyCIF image with selected markers shown; white dashed line separates the tumor and stroma compartment. **d** Adjacent H&E image of the same specimen as in panel (**c**). **e** Exemplary H&E image with individual tumor domains annotated with Gleason grades. **f** Infiltration of the B cells, CD4 T cells, and CD8 T cells per mm^2^ across Gleason grade groups G1–G5 (“3 + 3”, “3 + 4”, “4 + 3”, “4 + 4”, and “any 5”, respectively) within individually annotated tumor compartments (as in panel e) for all samples. The Jonckheere–Terpstra trend test (*P* value displayed) confirms an increasing lymphocytic immune-cell proportion with grade. Linear fit *P* values for each individual cell type are also shown (*F*-test). **g** Density of intratumoral CD20^+^ T cells (cells per mm^2^) by Gleason grade. The one-sided Mann-Whitney U test *P* value compares high- vs. low-grade disease; dashed lines indicate median reference densities in colorectal cancer as indicated. Point-wise one-sided Wilcoxon test *P* values for adjacent grade comparisons are indicated. **h** Similar to (**g**), density of intratumoral CD8^+^ T cells (cells per mm^2^) by Gleason grade. **i** Similar to (**g**), density of intratumoral CD4^+^ T cells (cells per mm^2^) by Gleason grade. **j** Regression fit and confidence intervals of the proportion of the intratumoral T cells (CD3d^+^) versus B cells (CD20^+^) stratified by Gleason status. *P* values represent the *F*-test of the fit.

To characterize the TME of PCa, we performed highly-multiplexed cyclic immunofluorescence (CyCIF) imaging^42^ on a retrospective cohort of 29 tumors of varying Gleason grade. These tumors were obtained from radical prostatectomies performed at Northwestern Memorial Hospital between 2005 and 2014. Patients were scored for Gleason grade, seminal vesicle involvement (SVI), perineural invasion (PNI), lymph node status, and tumor stage according to standard AJCC criteria (Fig. 1a). Some spatial analysis was performed using the Gleason grade of individual tumor regions as the independent variable (which varied from Gleason 3+3 at the lowest grade to regions scores as primary or secondary Gleason 5) but - consistent with clinical practice – most analysis used the whole tumor score defined as the maximum Gleason grade across histological sections. Whole tumor Gleason scores were dichotomized into “low Gleason grade” (LGG) corresponding to a maximal tumor Gleason grade of 3+4 and “high Gleason grade” (HGG) corresponding to Gleason 4+4 or any Gleason pattern 5. There were no patients with an overall Gleason grade of 4+3, although some tumor sub-regions exhibited Gleason 4+3 features. As expected, patients with high grade disease tended to have other high-risk clinical features, including lymph-node involvement, SVI, and PNI, although these associations were not statistically significant.

**Extended Data Fig. 1 |:**
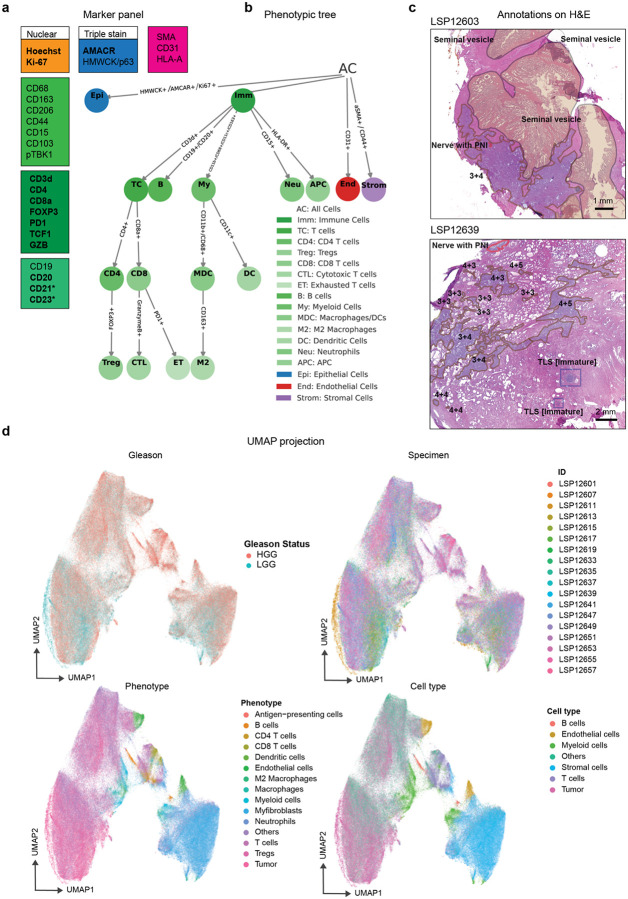
Cell phenotyping and quality control. **a** CyCIF markers grouped by function: epithelial markers (blue), stromal markers (pink), T-cell markers (red), innate-immune markers (purple), B-cell markers (green), and nuclear markers (orange). Key markers for this study are shown in bold and markers used specifically to study follicular dendritic cells in a subset of specimens are marked with an asterisk. **b** Phenotype hierarchy derived from the full marker set as used to assign cell types and states. **c** Examples of genitourinary (GU) pathologist annotations on H&E sections; two pathologists independently annotated the specimens, and their annotations were found to be in excellent agreement. **d** UMAP embedding of balanced sampling of 10,000 tumor and stromal cells per patient based on scaled, ungated marker intensities; points are colored by Gleason status, cell phenotype, and patient ID.

21-marker CyCIF^42^ spatial profiling was performed on whole slides (~10^6^ cells per specimen) rather than TMAs, providing the spatial power needed to characterize large immune clusters (IC)^43^ (Fig. 1b–c; Extended Data Fig. 1a; **Supplementary Table 1**). Images were processed using routines in MCMICRO^44^ to generate feature tables comprising the x-y coordinates of each segmented cell (2.7 × 10^7^ in total) and the mean intensities of each marker within either a nuclear or whole-cell segmentation mask. Per-cell marker intensities were gated to generate positive and negative classes (see Methods) and cell types were then identified based on binarized marker combinations (Extended Data Fig. 1b). To distinguish the tumor compartment and surrounding non-cancerous tissue, tumor boundaries were identified by board-certified pathologists using H&E images; most specimens were found to contain multiple tumor domains (1 to 13) separated by non-tumor prostate tissue ranging in size (in a 2D section) from as few as 44 cells in a tumor area of 0.0078 mm^2^ to 2.3 × 10^6^ cells in an area of 320 mm^2^ (Fig. 1d–e; Extended Data Fig. 1c); this corresponded to ~1 to 95% of the specimen area. Uniform manifold approximation and projection (UMAP) visualization on cell marker intensities (Extended Data Fig. 1d) confirmed the expected discrimination of immune, stromal and tumor cell types with specimen-level differences confined primarily to the tumor compartment.

**Extended Data Fig. 2 |:**
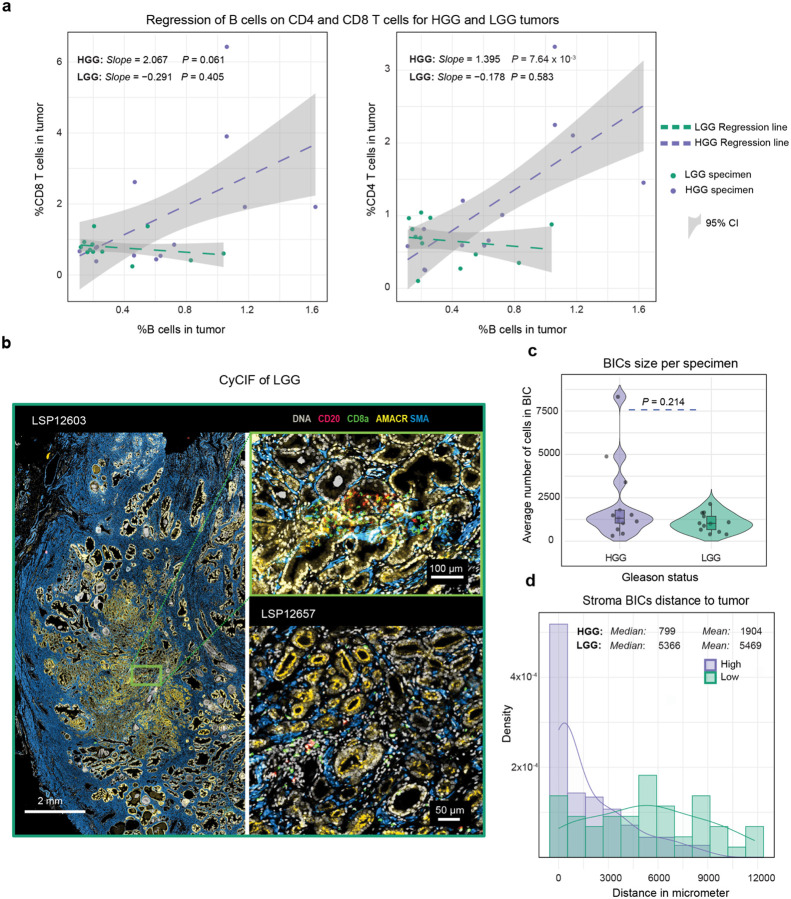
B and T cells densities and BICs exhibit distinct patterns between low - grade and high-grade PCa **a** Regression fit and 95% confidence intervals of the proportion of intratumoral CD8^+^ (left) and CD4^+^ (right) T cells compared with CD20^+^ cells by Gleason grade. Annotated values represent the *P* value of the fit (*F*-test) and the maximum likelihood estimate of the slopes. **b** Whole slide CyCIF image of a LGG case displayed with markers for nuclear DNA, smooth-muscle actin (SMA), CD8. Two zoomed-in exemplar regions are shown to the right. **c** Mean number of cells per BIC per tumor stratified by tumor grade, with *P* value representing Mann Whitney U test. **d** Histogram and smoothed curve of the tumor-BIC distances for extra-tumoral BICs, by tumor grade group.

When we quantified the proportions of segmented cells expressing the T cell markers CD4 or CD8, or B cell marker CD20 within histologically defined tumor domains, T cells were found to be ~8-fold more abundant than B cells. When individual tumor regions (99 domains total in 29 specimens) were stratified by Gleason grade group, we observed a monotonic increase in all three immune cell populations with increasing tumor grade (Jonckheere-Terpstra (JT) test, *P* = 0.008; Fig. 1f). The mean density of tumor-infiltrating CD20^+^ (B) cells varied almost 100-fold in the PCa cohort (~5 cells/mm^2^ to ~500 cells/mm^2^; Fig. 1g) and was significantly higher in HGG than LGG (Mann-Whitney U test, *P* = 0.006). As a reference, we compared immune cell densities in PCa to those in dMMR colorectal cancer (CRC)^45^, a classically immunologically “hot” tumor.^46^ In HGG PCa, B cell density was statistically indistinguishable from dMMR CRC, a B-cell rich tumor in which TLS abundance is predictive of slow tumor progression and ICI response. The density of CD3d^+^ CD8a^+^ cells (hereafter CD8 T cells) varied 38-fold greater across the PCa cohort (~17 to ~653 cells/mm^2^; Fig. 1h) but was not significantly different in LGG (mean = 71 cells/mm^2^) and HGG PCa (mean = 135 cell/mm^2^); it was also ~2–5-fold lower than either pMMR (254 cells/mm^2^) or dMMR (589 cells/mm^2^) CRC. While CD3d^+^ CD4^+^ cells (CD4 T cells) were significantly more prevalent in HGG than LGG PCa (Mann-Whitney U test, *P* = 0.046) they were also ~4-fold less dense than in CRC (Fig. 1i). The proportion of all CD3d^+^ cells (T cells) in HGG PCa tumor compartments was nonetheless positively correlated with the proportion of tumor-infiltrating B cells (*F*-test, *P* = 0.027) whereas there was no correlation in LGG tumors (*F*-test, *P* = 0.129; Fig. 1j). Similar results were obtained when relationships between B cells and either CD4 T or CD8 T cells was evaluated individually (Extended Data Fig. 2a). These results demonstrate that HGG PCa is infiltrated by B cells at a density similar to that of immunologically “hot” dMMR CRCs but has fewer T cells. Correlation in the levels of T and B cells in HGG PCa is nonetheless consistent with formation of organized ICs in which T and B cells interact.

### Immune clusters in high-grade PCa are more numerous and localized to the tumor region

**Fig. 2 |:**
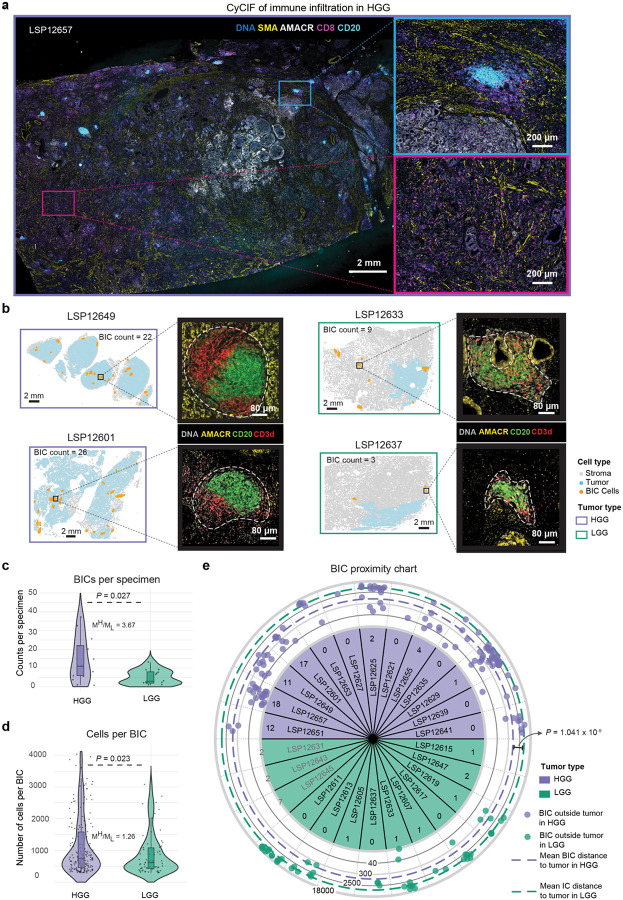
B-cell immune clusters (BICs) are larger, more numerous and more tumor-proximal in high-grade PCa **a** Whole-slide CyCIF image of an exemplar HGG case with high immune infiltration, displaying selected markers for nuclear DNA, smooth-muscle actin (SMA), CD20, and CD8. A region with a BIC and a region with dense CD8^+^ T cell infiltration is shown in light blue and purple windows, respectively. **b** Exemplar pseudo-images (cells represented as circles) for two HGG (left) and two LGG (right) specimens depicting non-tumor cells (gray), tumor region (light blue) and detected BICs (orange). CyCIF images with AMACR, CD20, and CD3d markers for selected BICs. The approximate boundary of each BIC is denoted by the white dashed line. **c** Number of the detected BICs per sample stratified by tumor grade. *P* values are from Mann-Whitney U test. The ratios of median values comparing HGG and LGG (M^H^/M_L_) are also shown. **d** Cell counts per BIC size stratified by tumor grade with similar test and comparison to (**c**). **e** Schematic depiction of the logarithmic distance from each BIC to the tumor compartment for extra-tumoral BICs (outer rings in μm), and the total count of intra-tumoral BICs for intratumoral BICs (inner ring). *P* value represents the Mann-Whitney U test of the BIC-tumor distance comparing HGG and LGG PCa. Three samples without annotated tumor regions on our slide are highlighted in grey.

Visual inspection of CyCIF images suggested that some B cells in HGG PCa were organized into compact ICs (Fig. 2a, Extended Data Fig. 2b) but not all B cell clusters had classical germinal center morphology as judged from adjacent H&E images. To identify B-cell enriched immune clusters (BIC) in an objective manner we used a K nearest neighbor (KNN) classifier with a minimum cluster size set empirically at 300 cells (see Methods). This yielded 257 BICs across 29 specimens. Some BICs had a compact round or ellipsoidal shape whereas others were less dense and more irregular; some BICs were located within the tumor domain and others lay in adjacent tissue (Fig. 2b; Methods).

We found that BICs in HGG were ~4-fold more abundant (median BICs per sample of 11.5 for HGG and 2.5 for LGG, Mann-Whitney U test, *P* = 0.027; Fig. 2c) and slightly larger than in LGG PCa (median cells per BIC of 750 for HGG and 610 for LGG, Mann-Whitney U test, *P* = 0.023, Fig. 2d; Extended Data Fig. 2c). In HGG PCa, BICs were twice as likely to reside within the tumor compartment as in LGG (Mann-Whitney U test, *P* = 0.019) and those BICs outside of the tumor compartment were ~7-fold closer to the tumor boundary in HGG than LGG (median tumor to BIC distance = 799 μm in HGG and 5366 μm in LGG; Mann-Whitney U test, *P* = 1.04 × 10^−9^; Fig. 2e; Extended Data Fig. 2d). Thus, BICs in HGG were both more frequent and more likely to be spatially associated with the tumor compartment than in LGG tumors. This suggests that BICs in PCa form in response to the presence of tumor and are not reflective of a generally inflamed stroma.

### Immune clusters in high-grade prostate cancer are organized and proliferative

**Extended Data Fig. 3 |:**
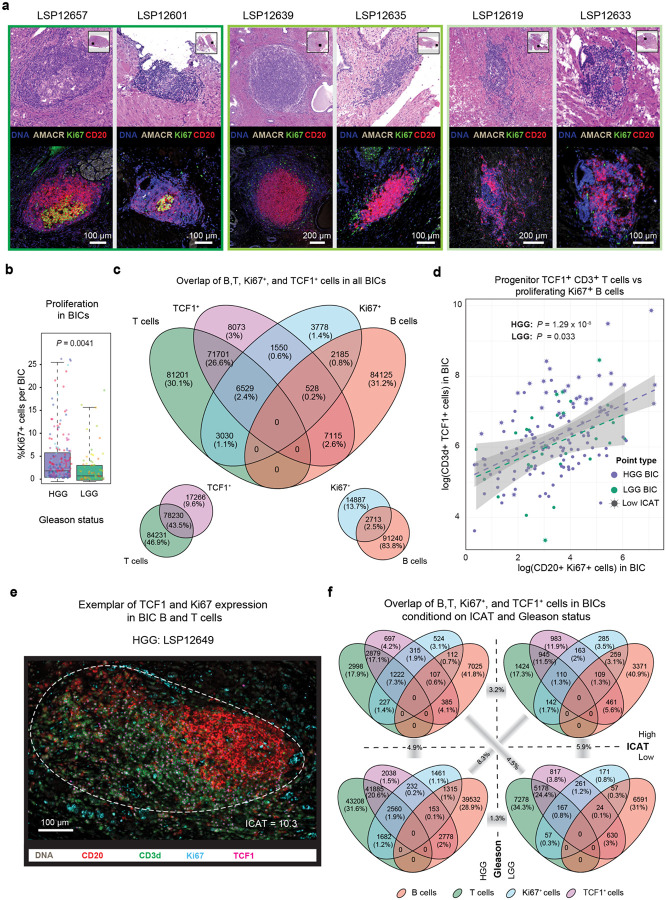
Spatially organized BICs contain Ki67^+^ proliferative cells and TCF1^+^ T cells. **a** Examples of BICs with mature TLS morphology (left), immature TLS morphology (middle) and disorganized morphology (right) based on expert H&E annotation and same-section identical magnification CyCIF. **b** Proportion of BIC cells that are Ki67^+^, colored by specimens and stratified by tumor grade (*P* value represents Mann-Whitney U test). **c** Venn diagram across all BICs showing the respective number and proportion of the cells for CD20^+^ (B cells), CD3d^+^ (T cells), TCF1^+^, and Ki67^+^. **d** The regression line and 95% confidence interval between the logarithmic scale of Ki67^+^ B and TCF1^+^ T cells within BICs, stratified by tumor grade. BICs with an ICAT in the lowest quartile (most organized; ICAT < 13.7) are shown with a star. *P* value represents *F*-test of the regression fit with *R*^2^_HGG_ = 0.22 and *R*^2^_LGG_ = 0.16. **e** CyCIF image depicting Ki67 and TCF1 signal on B and T cells in a BIC (encircled in white dashed line). **f** Venn diagrams in (**c**) stratified by ICAT score (top to bottom) and Gleason grade (left to right). The cross dissimilarities between each diagram’s proportion vector are measured by Jensen Shannon Divergence (JSD) reflected for the relevant pairs.

**Fig. 3 |:**
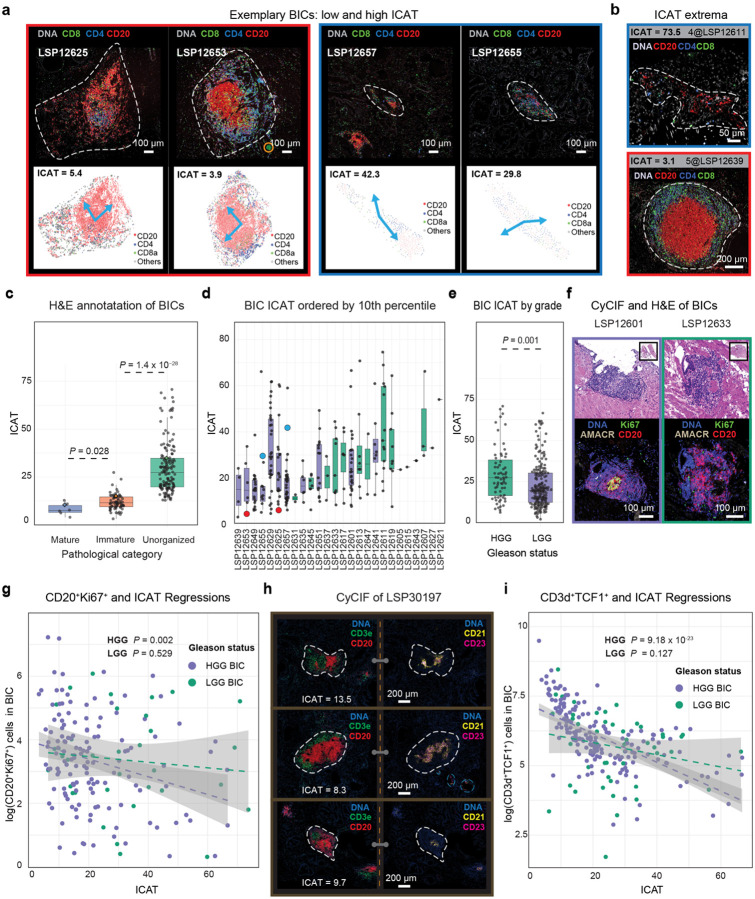
B cell clusters in high-grade PCa are spatially organized and harbor TLS-like immune-cells. **a** CyCIF (top; approximate BIC boundary represented by white dashed line) and pseudo-images (bottom; cells are circles) of two highly organized (left panel) and disorganized (right panel) BICs. The blue arrows represent the independent components as determine by ICA analysis. Image artefacts are circled in orange. **b** CyCIF images of the BICs with the highest (top; least organized) and lowest ICAT values (bottom; most organized). **c** BIC ICAT scores stratified by pathologist classification. *P* values represent Mann-Whitney U tests. **d** Distribution of ICAT values per sample, ordered by 10^th^ percentile of ICAT score. The four BICs in (**a**) are marked with red and blue for the left and right panel, respectively. **e** Distribution of ICAT values by high-grade (HGG) versus low-grade (LGG), shown with *t*-test *P* value. **f** H&E (top) and CyCIF (bottom) of exemplar BICs with (left) and without (right) a Ki67^+^ proliferative germinal center. **g** Regression fit and 95% confidence interval of proliferative B cells (CD20^+^ Ki67^+^) compared with ICAT values per BIC with *R*^2^_HGG_ = 0.07 and *R*^2^_LGG_ = 0.01 and the *F*-test *P* value for regression fits. **h** CyCIF images of three TLSes as characterized with follicular dendritic markers CD23 and CD21, depicted with the corresponding ICAT values for an external sample (LSP30197). Image artifacts are encircled with dashed light blue lines. **i** Regression fit and 95% confidence interval of TCF1^+^ T cells (CD3d^+^ TCF1^+^) compared with ICAT values per BIC with *R*^2^_HGG_ = 0.49 and *R*^2^_LGG_ = 0.05 and the *F*-test *P* value for regression fits.

To quantify differences in BIC morphology and density (Fig. 3a) we developed an approach based on independent component analysis (ICA)^47^; this mapped the positions of specific cell types (in the current case, CD20^+^ B cells) onto maximally independent axes of dispersion such that the linear correlation between the cells in x-y coordinates tended to zero (see Methods). The normalized trace of the standard deviation matrix of the independent components (ICAT) then quantified the spatial dispersion, with low values associated with dense and symmetric BICs, and high values with BICs less dense in immune cells and having irregular shapes (Fig. 3a–b, Methods, Supplementary Data S1). We validated the ICAT metric using expert visual review of adjacent H&E images. Among 257 BICs, six (2.2%) were judged from H&E images to have a high likelihood of representing a mature TLS with a germinal center; all were intra-tumoral BICs from HGG cases. An additional 72 (27%) of BICs were classified as immature TLS, and 65/72 of these (90%) were found in HGG tumors. The remaining 181 BICs were classified as disorganized immune clusters with atypical and sparse cellular aggregates (Extended Data Fig. 3a). ICAT values recapitulated manual classification: mature BICs corresponded to ICAT= 8.12 ± 3.2, immature BICs to ICAT = 11.92 ± 4, and disorganized BICs to ICAT = 29.99 ± 13.8 (Kruskal-Wallis test of similarity, *P* = 2.2 × 10^−16^; Fig. 3c). As judged visually from H&E images, mature or immature TLS were ~4-fold more prevalent in HGG than in LGG PCa and BICs from HGG PCa were associated with significantly lower ICAT values than BICs from LGG tumors (Mann-Whitney U test, *P* = 0.001; Fig. 3d–e). We therefore conclude that BICs in HGG PCa are significantly more organized than those in LGG PCa and that greater organization corresponds to the histopathological definition of “maturity” for an IC.

TLSs are organized ICs in which B cells undergo selection, amplification, affinity maturation, and eventual production of antibodies against tumor antigens^48^. A common measure of this process is the presence of proliferating Ki67^+^ B cells^49^. Across all BICs, we observed that ~3% of B cells were Ki67^+^. However, BICs in HGG exhibited a significantly increased Ki67^+^ proportion as compared to LGG BICs (Fig. 3f, Extended Data Fig. 3b). A significant negative relationship was also observed between the log number of Ki67^+^ B cells and ICAT values in HGG tumors themselves (*F*-test, *P* = 0.002 and *P* = 0.529 for HGG and LGG, respectively; Fig. 3g). Moreover, detailed CyCIF characterization using an expanded TLS-focused panel (**Supplementary Table 1**) of an HGG PCa having ICs with ICAT value in the lower quartile (< 13.7 = Q_1_, corresponding to mature and immature histological classes; Fig. 3h) showed that organized BICs contained foci of CD21^+^ and CD23^+^ follicular dendritic cells (FDCs). The presence of FDCs is a key feature of functional germinal centers. We conclude that low ICAT organized ICs are more likely to have proliferating B cells and FDC foci, two characteristics of functional TLS.

TLSs also promote programming and amplification of “stem-like” T cells, which can be identified based on expression of the TCF1/TCF7 transcription factor^50^. BICs in our PCa cohort contained ~1.3-fold as many T as B cells and 48% of these T cells were TCF1^+^ (among these, 8% were also Ki67^+^, Extended Data Fig. 3c). The log-number of TCF1^+^ T cells was negatively correlated with ICAT values for the HGG group (*F*-test, *P* = 9.18 × 10^−23^ for HGG and *P* = 0.127 for LGG; Fig. 3i). The number of Ki67^+^ B cells and TCF1^+^ T cells was positively correlated in log-log space (*F*-test, *P* = 1.29 × 10^−8^ for HGG and *P* = 0.033 for LGG). Another way of viewing this result is that Ki67^+^ B and TCF1^+^ T cells were highly enriched in BICs having ICAT values less than its 1^st^ quartile (Q_1_ = 13.7) (Extended Data Fig. 3d–e), and thus, segregation on ICAT interquartile value alone (<Q_1_ = 13.7 and >Q_3_ = 32.4) was effective at discriminating BICs with proliferating B cells and stem-like T cells in both HGG and LGG PCa. (Extended Data Fig. 3f). These data show that BICs in HGG are not only more numerous than in LGG PCa, but that they were significantly more organized (having lower ICAT values). This higher degree of organization was associated with a greater prevalence of proliferating B cells and stem-like T cells. This correlation between Ki67^+^ B cell and TCF1^+^ T cell prevalence in a subset of well-organized ICs is consistent with a dynamic and active immune response involving both B and T cells.

### High-grade prostate cancers exhibit dense T-cell clusters closely associated with B-cell aggregates

**Fig. 4 |:**
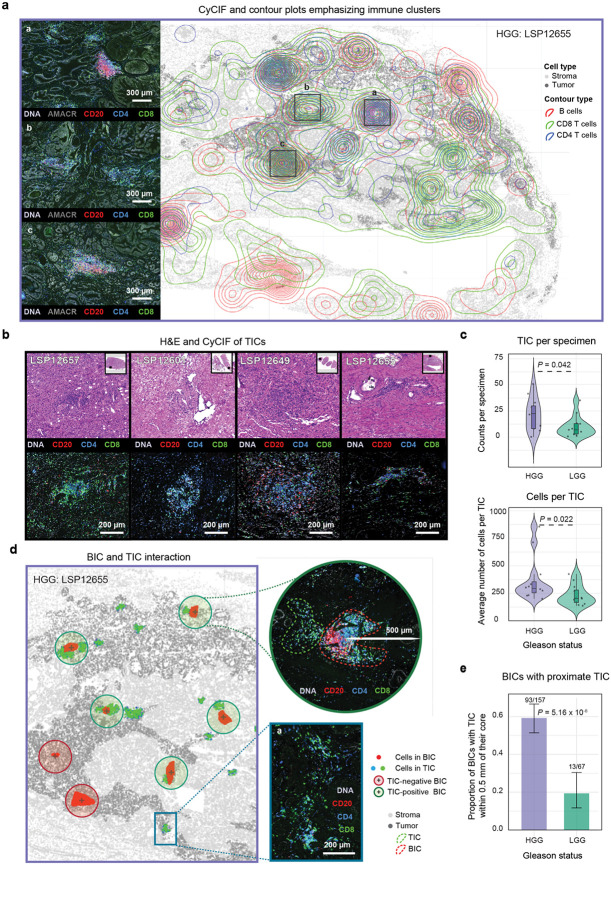
T cell clusters (TICs) are abundant and spatially co-localized with B cell clusters in high-grade PCa. **a** Contour plot of an HGG specimen with high CD20^+^ and CD4^+^-CD8^+^ density areas (marked as *a* and *c*) and dense areas of CD4^+^ and CD8^+^ without apparent CD20^+^ intensity (marked as *b*). **b** Examples of four post-CyCIF H&E images (top) and CyCIF images (bottom) denoting the T cell- clusters (TICs). **c** Mean cell counts (top) and number (bottom) of TICs per sample, stratified by tumor grade (*P* values represent Mann-Whitney U test). **d** Pseudo-image (cells are dots) of a HGG specimen depicted with BICs (red) and TICs (green), overlaid with green and red circled area denoting BICs with (green) or without (red) TIC within 500 μm from BIC core. Shown are exemplar corresponding CyCIF images with BIC (red dotted line) and TIC (green dotted line) circled (top) and a TIC without a corresponding BIC and particular organized shape (panel *a* bottom). **e** Bar plot and 95% confidence interval of the proportion of BICs proximal a TIC (500 μm), stratified by tumor grade (*P* value denotes proportion test).

The analysis of ICs described above focused on B cells because these are key constituents of germinal centers; however, inspection of images also revealed the presence of primarily T cell aggregates. As a first step in evaluating the extent of B and T cell co-clustering, we generated CyCIF contour density maps for B, CD4, and CD8 T cells (Fig. 4a). This revealed the presence of T cell-rich immune clusters lacking B cells (TICs; Fig. 4a panel b) as well B-cell specific BICs and ICs in which B and T cells were coincident (as described above). Examination of TICs in adjacent H&E images showed that they corresponded to lymphocytic aggregates that were indistinguishable by human inspection from less organized BICs (Fig. 4b, Extended Data Fig. 3a). To enumerate and characterize TICs, we performed DBSCAN (Density-Based Spatial Clustering of Applications with Noise) on CD4 and CD8 T cells (Methods)^51^. DBSCAN is a non-parametric density-based clustering algorithm that groups points (cells in this case) that meet a specific density per unit area criteria. It is effective at identifying clusters with varying geometries and lack of clear centrality, which was typical of TICs in PCa (Fig. 4b & d; panel a).

**Extended Data Fig. 4 |:**
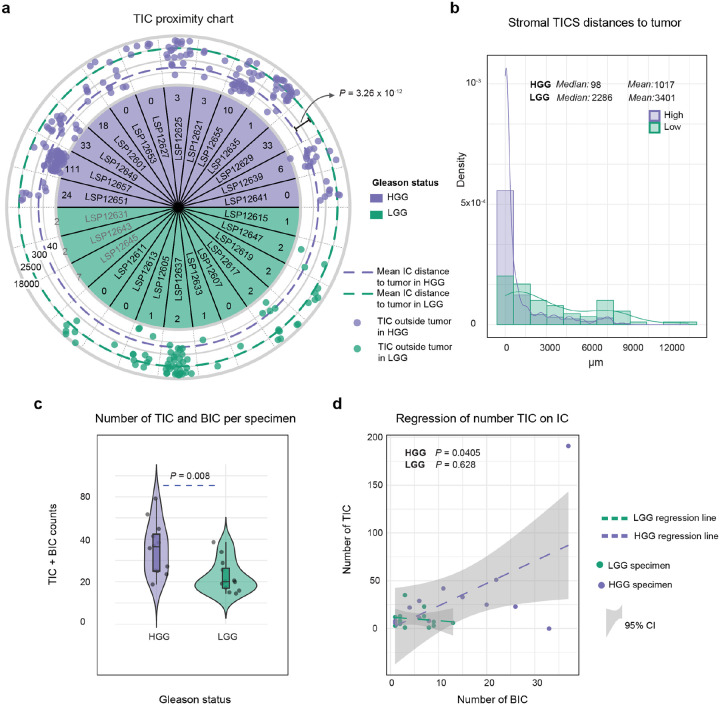
T cell immune clusters are more numerous and more spatially proximal to tumors in high-grade PCa. **a** Schematic depiction of the logarithmic distance from each T cell immune cluster (TIC) to the tumor compartment for extra-tumoral TICs (outer rings in μm), and the total count of intra-tumoral TICs for intratumoral TICs (inner ring). *P* value represents the Mann-Whitney U test of the TIC-tumor distance comparing HGG and LGG PCa. Three samples without annotated tumor regions on our slide are highlighted in grey. **b** Histogram and smoothed curve of the tumor-TIC distances for extra-tumoral BICs, by tumor grade group. **c** Total number of BICs and TICs in each specimen stratified by tumor grade (*P* value represents Mann-Whitney U test). **d** Regression fit and 95% confidence interval for the number of TICs compared with number of BICs, stratified by tumor grade (*P* values represent *F*-test of the fit).

DBSCAN identified 588 TICs across the PCa cohort, and these were on average ~3-fold smaller (median 213 cells per TIC v. 713 cells per BIC; Mann-Whitney U test, *P* = 0.026) and 4 times “less organized” than BICs as measured by ICAT value (median TIC ICAT = 94; median BIC ICAT = 22, Mann-Whitney U test, *P* = 2.2 × 10^−16^). TICs were significantly more abundant and larger in HGG as compared to LGG tumors (median of 7 TICs per specimen of 174 cells each in LGG vs. 24 TICs per specimen of 272 cells each in HGG; Mann-Whitney U test, *P* = 0.042 and *P* = 0.022, respectively; Fig. 4c) and were more likely to lie within or immediately adjacent to the tumor domain (HGG vs. LGG, Mann-Whitney U test, *P* = 3.26 × 10^−12^; Extended Data Fig. 4a–c). Thus, HGG PCa has larger and more organized TICs than LGG PCa, some of which are spatially associated with BICs.

Interaction of T and B cells is necessary for antigen presentation by B cells to helper T cells, co-stimulatory signaling, cytokine exchange, and the formation of immune synapses that facilitate B cell maturation and T cell activation^52–54^. Spatial proximity of B and T cell clusters is therefore reflective of an active immune response. In PCa we observed that the numbers of BICs and TICs per specimen were positively correlated in HGG (*F*-test, *P* = 0.04) but not LGG (*F*-test, *P* = 0.63; Extended Data Fig. 4d). In a mature TLS, B and T cells are not fully intermixed however, but instead exhibit “zonation”; this typically manifests itself as a T cell crescent surrounding a B cell core^55^. We observed that the proportion of BICs with a TIC within 500 μm of the BIC center was 3-fold higher in HGG than LGG tumors (~60% vs. ~20%; two-proportion z-test, *P* = 5.6 × 10^−8^; Fig. 4d–e). Thus, TICs in HGG are more abundant, larger in size, and more likely to be associated with BICs.

### B and T cell spatial organization in prostate cancer extends beyond discrete clusters

**Extended Data Fig. 5 |:**
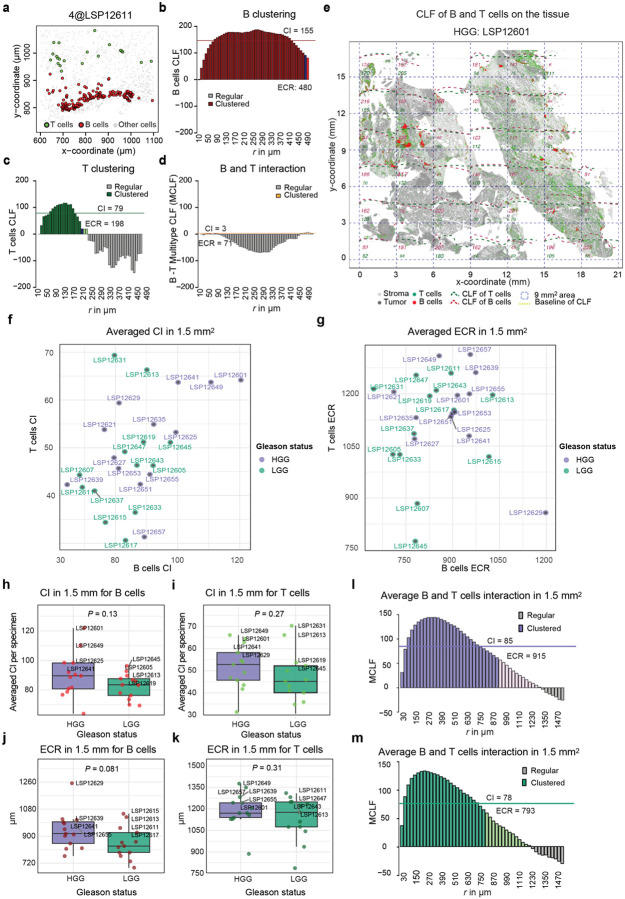
The spatial interaction of B and T cells across whole-slide prostate cancer images. **a** Pseudo-image (cells are circles) showing B and T cell communities on the tissue in a LGG case. **b** The corresponding centered L-function (CLF) for B cells, highlighting a consistently higher clustering intensity (CI) over a larger radius (ECR). **c** The CLF for T cells, indicating a phase shift toward complete spatial randomness (CSR) and regularity, with lower CI and ECR. **d** The interaction between B and T cells, characterized by the multitype CLF (MCLF), underscoring consistent regularity indicative of complete segregation. **e** Depiction of whole-tissue B (CD20^+^) and T (CD3d^+^) cells, along with a clustering survey using the centered L-function (CLF) over 9 mm^2^ square areas, binned over increasing 30-μm radii (dashed curves), with marked positive integral values in red and green for each area, respectively. **f** Point plot of clustering intensity (CI) for B and T cells, stratified by Gleason status, with annotations for specimen identifiers. **g** Point plot of effective clustering radius (ECR) for B and T cells, stratified by Gleason status, with annotations for specimen identifiers. **h** CI for B cells, stratified by Gleason status, showing respective comparison Mann-Whitney U test *P* values, with annotations for the four highest values in each plot including specimen identifiers. **i** Similar to (**h**), boxplots for T cells. **j** Similar to (**h**) boxplots for ECR. **k** Similar to (**h**) boxplots for T cells and ECR. **l** Multitype CLF (MCLF) depicting the interaction between B and T cells, integrated across all fourfold systematic bootstrapped 9 mm^2^ square areas for each specimen and averaged for each 30-μm radius bin in HGG samples, overlaid with co-clustering intensity (CCI) and estimated co-clustering radius (ECCR). **m** Multitype CLF (MCLF) depicting the interaction between B and T cells, integrated across all fourfold systematic bootstrapped 9 mm^2^ square areas for each specimen and averaged for each 30-μm radius bin in LGG samples, overlaid with co-clustering intensity (CCI) and estimated co-clustering radius (ECCR).

**Fig. 5 |:**
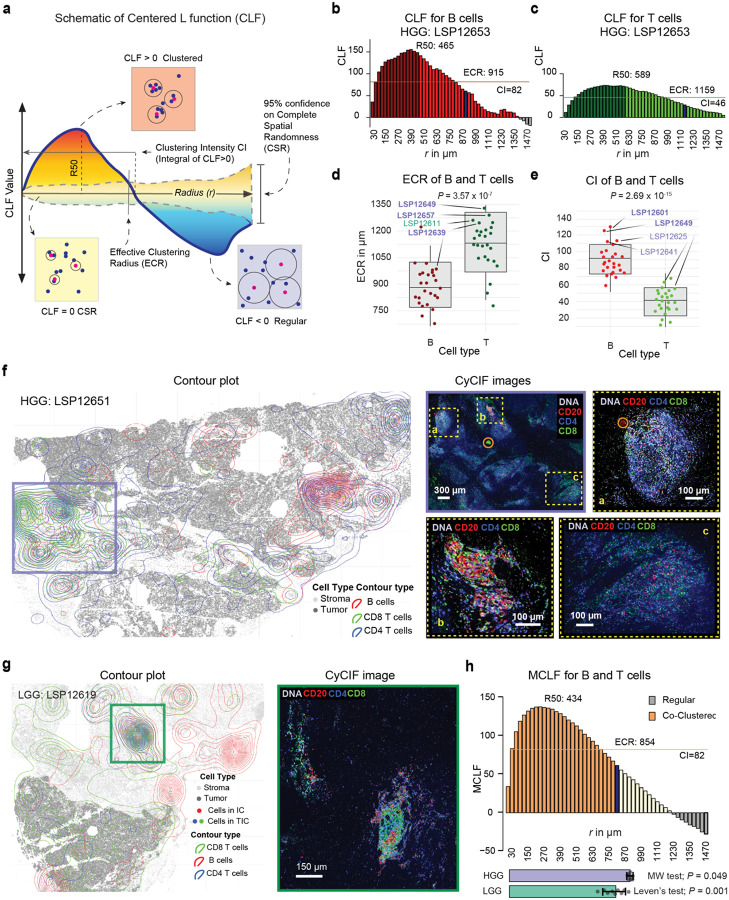
HGG exhibit increased B and T cells interaction on larger length scale. **a** Schematic depiction of the centered L-function (CLF) over a span of radii (*r*), showing the complete spatial randomness (CSR) line and the expanding confidence interval around it. As a function of *r*, the example CLF begins with mild clustering at small radii, peaks at extreme clustering near R50 radius, crosses the CSR line into regularity in the CSR region, and then at higher radii exhibits non-random anti-clustering (regularity), returning to the CSR confidence area; R50: the maximum radius below which 50% of the positive integral accumulates, effective clustering radius (ECR): the maximum radius where significant positive clustering exists. **b** The averaged CLF for B cells in 9 mm^2^ areas on the tissue, using fourfold systematic bootstrapping, showing peak clustering at radius of 455 μm. **c** Similar CLF and indices as in (**b**), but for T cells. **d** ECR between T and B cells across the cohort (*P* value represents Mann Whitney U test). The four highest values in each boxplot are annotated with specimen identifiers for HGG (purple) and LGG (green), with specimens in the top 10th percentile for infiltrating CD20^+^ or CD8^+^ cells in the tumor across the cohort shown in bold. **e** Clustering intensity (CI) between T and B cells across the cohort, with similar annotations as in (**d**) but *t*-test. **f** Contour plot of an exemplar HGG sample highlighting large B and T cell co-clustering areas, with an overview of the CyCIF image area and zoomed-in sub-areas colored by CD20, CD4, and CD8 markers. Image artifacts are circled in orange. **g** Contour plot of an LGG sample with an enlarged CyCIF image area showing a dense T cell community. **h** The integrated multitype CLF (MCLF) as an average co-clustering survey of the whole cohort on 9 mm^2^ areas, depicting the interaction function for co-clustering (or co-regularity) of B and T cells similarly to (**b**), with overlaid co-clustering intensity (CCI) as the integral of the positive area of the MCLF and estimated co-clustering radius (ECCR) as the maximum radius where significant positive co-clustering between B and T cells exists. The horizontal bar plot at the bottom shows the average ECCR for HGG and LGG samples with respective 95% confidence intervals, with differences in means and variances quantified by *P* values from the Mann-Whitney U test and Levene’s test, respectively.

As a complementary approach to quantify B and T cell distributions across tumor and stroma compartments, we used the centered L-function (CLF). This function quantifies the degree of spatial interaction in sets of objects (different cell types in this case) at varying radii (Supplementary Data S2, Fig. 5a) by computing the degree of divergence from complete spatial randomness (CSR) while accounting for differences in the intensity of the points (cells per unit area). Divergence can either be positive (upward from the X axis, with CLF=0 corresponding to CSR), representing greater clustering (red region in Fig. 5a) or negative, representing regularity (blue region) (Methods). The CLF distribution can be parameterized by a clustering intensity (CI), which is the integral of positive values of the function and an effective clustering radius (ECR) which is the point at which the CLF reaches zero and complete spatial randomness is reached. To make this estimate robust, the ECR is defined as the point at which the CLF crosses the upper ninety-five percent confidence interval for the estimate of CLF= 0 (Fig. 5a, Extended Data Fig. 5a–d, Methods, Supplementary Data S2). In contrast to the clustering methods described earlier, the CLF is ideal for quantifying the spatial distributions of cells irrespective of their density, and the ECR provides a robust estimate of the radius (*r*) up to which a significant deviation from randomness toward clustering is observed.

For optimal detection of mesoscale structures, we computed the CLF by applying it to whole-slide images using a 9 mm^2^ sliding square window. This window size was selected based on exploratory studies that showed it to be ~3 times greater than the largest ECR (Extended Data Fig. 5e, Methods). The CLF was summed across these windows to generate a per-tissue parameterization of immune cell organization; functions were also averaged across the cohort to generate summary statistics (Methods). By way of example, for HGG specimen LSP12653 (Fig. 5b–c), the B cell CLF rose to 150 at a radius of ~455 μm and then fell monotonically to CSR at ~1.1 mm, yielding a CI of 82 and ECR for B cells of 915 μm (Methods, Supplementary Data S2). For T cells, the CLF was similar in shape, yielding a CI of 46 and ECR of 1159 μm. Across the cohort, B cells in HGG samples exhibited significantly greater CLF clustering intensity (mean CI = 92) over a shorter distance (mean ECR = 864 μm) than T cells (mean CI = 42, mean ECR = 1129 μm; Mann-Whitney U test, *P* = 2.69 × 10^−15^ and 3.57 × 10^−17^, respectively; Fig. 5d–e; Extended Data Fig. 5f–k). These radii are ~4 to 8-fold greater than the median radii for ICs obtained using KNN and DBSCAN clustering methods (~280 & ~140 μm, respectively) and demonstrate that B and T cells in PCa are non-randomly distributed outside of the relatively compact structures that define the “cores” of BICs and TICs (Fig. 2a–b, 3a, 4a–b).

To quantify co-clustering of T and B cells at diverse length scales (Fig. 5f–g), we applied the multitype CLF (MCLF), which quantifies the spatial distribution of cell - cell interactions (Methods, Supplementary Data S2, Extended Data Fig. 5d). When MCLF parameters were compared for HGG and LGG tumors we observed greater co-clustering radius in the former with tighter standard deviation (ECCR = 915 vs. 793 μm, Mann-Whitney U and Leven’s test, *P* = 0.049 & *P* = 0.001, Fig. 5h, Extended Data Fig. 5l–m). Thus, B and T cells are more likely to interact over longer spatial scales in HGG than LGG tumors and the region of interaction extends beyond the IC cores. These findings further confirm that HGG has a more spatially organized distribution of B and T than LGG and that non-random distributions of B and T cells can be detected at both short and long radii (from single cell diameters to *r ~*1 mm), potentially reflecting a diversity of functional interactions.

### Evidence of active immunosurveillance and potentially targetable T cell programs in PCa

**Extended Data Fig. 6 |:**
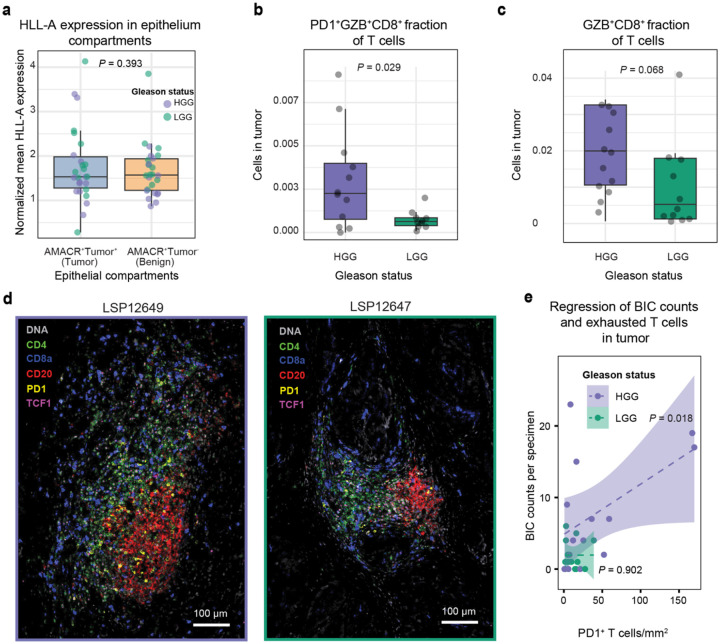
Further quantification of T cell subtypes and HLA-A expression in HGG versus LGG PCa. **a** Mean normalized HLA-H immunofluorescence expression in cells from the non-tumor and tumor compartments of the tissue (*P* value represents paired *t*-test). **b** Proportion of PD-1^+^ GZB^+^ CD8^+^ cells among T cells in the tumor for HGG and LGG tumors (*P* value represents Mann-Whitney U test). **c** Proportion of GZB^+^ CD8^+^ cells among T cells in the tumor for HGG and LGG tumors (*P* value represents Mann-Whitney U test). **d** Portion of CyCIF images denoting increased expression of PD-1 and TCF1 around the BIC in two HGG specimens. **e** Regression fits of the number of BICs in each specimen against the density of PD-1^+^ T cells in the tumor, stratified by HGG and LGG tumors, with corresponding *F*-test *P* values for the fits and 95% confidence intervals.

**Fig. 6 |:**
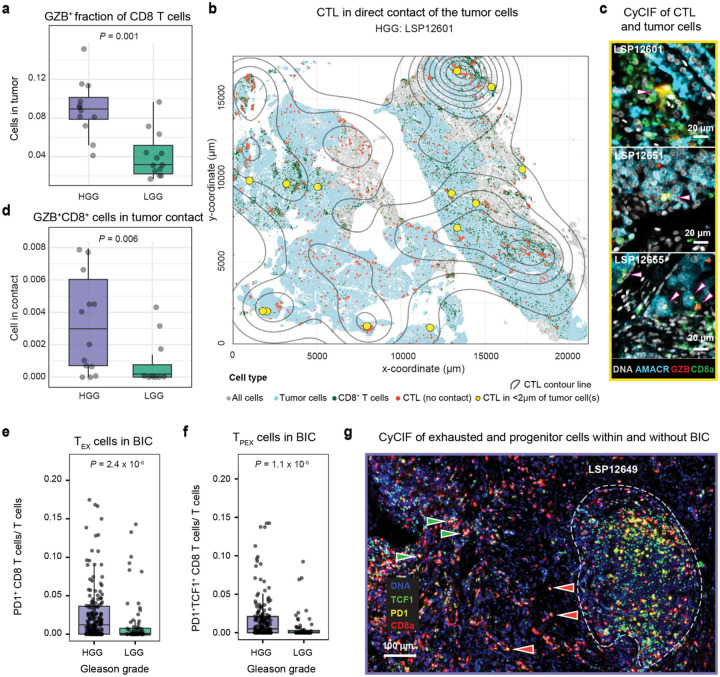
High-grade PCa exhibits spatially restricted enrichment of stem-like T cells and dendritic cells in B cell clusters. **a** Cytotoxic T cell (CTL; GZB^+^ CD8^+^) proportions in the tumor for HGG and LGG samples (*P* value represents Mann-Whitney U). **b** Pseudo-image of an HGG sample showing the tumor region, CD8^+^ T cells, and CTLs. CTLs in contact with tumor cells (<2 μm) are shown in yellow, with contour lines depicting the spatial frequency of CTLs. **c** CyCIF images of CTLs in contact with tumor cells, indicated by overlaid arrows. **d** Proportion of CTLs in contact with tumor cells (<2 μm) for HGG and LGG samples (*P* value represents Mann-Whitney U test). **e** Percentage of T_EX_ exhausted CD8 T cells (PD-1^+^ CD8^+^) and **f** T_PEX_ progenitor-exhausted cells (TCF1^+^ PD-1^+^ CD8^+^) relative to total T cells (CD3d^+^) within BIC regions, stratified by high-grade (HGG, purple) and low-grade (LGG, green) tumors (*P* value represents Mann-Whitney U test). **g** Representative CyCIF image highlighting increased exhaustion and progenitor-exhausted cell states adjacent to a BIC. TCF1^+^ PD-1^+^ CD8 T cells and TCF1^−^ PD-1^+^ CD8 T cells are indicated by right-pointing green arrows and left-pointing red arrows, respectively. The BIC area is encircled by a white dashed line.

Effective T cell mediated immune surveillance requires antigen presentation by tumor cells and interaction with cytotoxic T cells (CTLs). Loss of HLA class I expression is a common route of escape from immune surveillance. We compared normalized HLA-A expression in AMACR-positive tumor cells with the matched AMACR-positive benign epithelium from the same sections (as a control) but found no significant difference (paired *t*-test, *P* = 0.39, Extended Data Fig. 6a). CTLs can be identified in CyCIF data based on expression of the cytolytic protease granzyme B (GZB) and we found that CD8^+^ PD-1^+^ GZB^+^ cells comprised up to 0.9% of all T cells in PCa specimens and were almost 2-fold more abundant in HGG than LGG PCa (median of total number of CTLs divided by all T cells of ~3 × 10^−3^ and ~4 × 10^−4^, respectively; Mann-Whitney U tests, *P* = 0.029, Extended Data Fig. 6b). The GZB^+^ fraction of CD8 T cells and CD8^+^ GZB^+^ of the T cells were significantly higher in HGG as compared to LGG (Man-Whitney U test, *P* = 0.001 and *P* = 0.068, Fig 6a; Extended Data Fig. 6c). When we scored the proximity of the latter to the tumor cells using a stringent interaction distance of ≤ 2 μm, we observed a significant difference between HGG compared to LGG PCa (Mann-Whitney U test, *P* = 0.006. Fig. 6b–d). These data suggest that HGG PCa contains CTLs, a subset of which are in proximity to tumor cells, and that these tumor cells express HLA-A and potentially other Class I molecules at normal levels. Thus, it seems likely that tumors, particularly in HGG PCa, are subject to active immune surveillance by T cells.

TLS have been shown, in some cancers, to play a role in responsiveness to PD-1 and PD-L1-directed immunotherapies^31,56^. Whereas antigen-experienced CD8^+^ PD-1^+^ cells can be found both in TLS and in the tumor compartment^50^, PD-1^+^ TCF1^+^ CD8^+^ T cells (which likely corresponded to “precursor of exhausted” or T_pex_ cells)^50^ are generally enriched in TLS, where they interact with proliferating B cells (Extended Data Fig. 6d). In our cohort, we found that CD8^+^ PD-1^+^ T cells comprised a ~5-fold greater proportion of all T cells in BICs from HGG than LGG PCa (Mann-Whitney U test, *P* = 2.4 ×10^−6^, Fig 6e; Extended Data Fig. 6e) and that T_pex_ cells were ~7-fold more prevalent in HGG BICs (Mann-Whitney U test, *P* = 1.1 × 10^−6^, Fig. 6f). In contrast, no significant difference in the abundance of PD-1^+^ CD8^+^ cells was observed outside of BICs with or without stratification on TCF positivity (Mann-Whitney U test, TCF1^−^: *P* = 0.11 and TCF1^+^: *P* = 0.19, Fig. 6g). These findings show that HGG prostate cancer exhibits significantly higher proportions of T_pex_ cells within BICs compared to LGG, highlighting the potential role of the TLS in supporting therapeutically targetable T cell programs.

## DISCUSSION

In this study we quantify the composition and spatial organization of immune complexes in primary PCa, with an emphasis on high-grade tumors that account for a disproportionate share of overall mortality^40,41^. Both metastatic and primary PCa are often considered immunologically “cold”^57^ cancers, in part because ICIs have not proven effective in these settings in unstratified trials. However, we find that a substantial fraction (~30–40%) of HGG PCa (Gleason grade groups 4 and 5) is as highly infiltrated with B cells as dMMR CRC cancer, an archetype of an immunologically hot cancer. T cells are ~4-fold less abundant (per unit area of tumor) in PCa than dMMR CRC, but they are nonetheless organized into immune complexes with the hallmarks of an active immune response. A subset of effector CD8 T cells is also PD-1 positive, expresses GZB, and is in intimate contact with tumor cells, all features of anti-tumor cytotoxicity^58^. The TCF1^+^ PD-1^+^ CD8^+^ cells present in HGG ICs are likely to represent precursor exhausted (T_PEX_) T cells, which are increasingly recognized as critical for responsiveness to ICI therapies: T_PEX_ cells retain proliferative potential and the ability to differentiate into effector cytotoxic T cells following PD-1/PD-L1 blockade^59,60^. A key conclusion from this study is therefore that HGG PCa is not immunologically cold: it contains abundant B and T-cell focused immune clusters, stem-like T cells, and CTLs in the tumor compartment itself.

### Quantifying and characterizing immune clusters

A hallmark of an active immune response is coordination among diverse cell types through cell-cell signaling and spatial clustering. The best organized ICs act as hubs for antigen presentation, T-cell co-stimulation, B-cell activation and affinity maturation, as well as other local anti-tumor responses^61^. In multiple tumor types^30^ the presence of TLSs^31,49,56,62,63^ is associated with improved clinical outcomes as well as benefit from immunotherapy^31,56^. Prior H&E and IHC studies have suggested that immune cells in PCa are organized spatially, but the absence of an objective means of identifying and classifying ICs has impeded analysis. We found that classical germinal center morphology, as judged by trained histopathologists from H&E images^64,65^, is present on a minority of ICs in PCa, but that many ICs lacking classical morphology were significantly non-random and had molecular features associated with TLS. We therefore used a set of simple statistical methods to generate a rigorous but intuitive picture of these immune cell distributions and their relationship to tumor grade; these approaches are likely to be broadly useful for other cancer types and biological variables.

A K nearest neighbor (KNN) algorithm was used to identify and locate B-cell enriched BICs. Starting with a cell centroid, KNN classifies cells based on the dominant property of its K nearest neighbors (K is the number of nearest neighbors and was set to 100 in the current study, although this did not prove to be a sensitive parameter). KNN clustering was most effective with relatively dense and uniform clusters (typically ≥50% of the 100 nearest having the same marker label) and identified 257 BICs across 29 specimens, with significantly more in HGG than LGG PCa. Visually, the distribution of T cells was more diffuse than that of B cells, and KNN clustering missed some of these aggregates. DBSCAN, an unsupervised method that groups together cells with similar marker labels and is robust to noise and outliers was effective in this setting and detected 588 TICs, many of which were coincident or proximate to BICs, with significantly greater prevalence in HGG than LGG PCa. TICs contained ~3-fold fewer cells than BICs and were generally less densely packed, likely explaining why DBSCAN was more effective than KNN clustering in TIC detection. A complementary approach we used the centered L-function (CLF) to detect and parameterize significant deviation in cell distribution from complete spatial randomness; in PCa this demonstrated non-random T and B distributions (and co-occurrence) at radii as great as ~1 mm, a value 2–4-fold greater than cluster radii obtained by KNN or DBSCAN. Assemblies this large cannot be characterized accurately using tumor microarrays (TMAs): it is not possible to accurately enumerate and characterize structures such as an IC whose length scales are similar to or larger than the viewing window, emphasizing the necessity of whole- slide analysis^43^.

As a summary spatial statistic for characterizing within and across specimens we used independent component analysis^47^, a classical dimensionality reduction related to principal component analysis (PCA). ICA separates multivariate signals (the spatial distributions of cells in the current case) into statistically independent but not necessarily Gaussian components and ICAT, the normalized trace (the sum of the diagonal elements) of the ICA standard deviation matrix captures the degree of symmetry and the density of ICs in a single parameter. Direct comparison of ICAT values with expert pathology annotation of H&E images confirmed their correspondence and showed that BICs and TICs were significantly more organized in HGG than LGG PCa. ICs with a high degree of organization (a low ICAT value) were more likely to exhibit features of germinal centers involved in B cell amplification and T cell programming including proliferating B cells, TCF1^+^ T cells, and CD21^+^ and CD23^+^ follicular dendritic cells. However, only a minority of these ICs had classical germinal center morphology in H&E images suggesting that this is too strict a criterion for ICs in which B and T cells have the potential to interact functionally^48^.

### Diagnostic and therapeutic implications

Our findings are consistent with a recent study^66^ that used machine learning on a large set H&E images in combination with bulk RNA-Seq of tumors (n ~ 300 to 500 patients) to show that clustering of immune cells in high grade PCa is associated with reduced risk of distant metastasis. Cancers with many ICs in that study were also enriched in the CD8 and CD4 cells. Thus, the type of immune profiling described here may have a role in risk stratification of patients with localized but high-grade disease. Our findings also contribute to accumulating evidence that, despite historically low response rates to ICIs in mCRPC, it may be appropriate to revisit immunotherapy in selected primary PCa patients^67,68^. One possibility is that selection for tumors enriched in CD8^+^ T cell infiltration, as currently being explored for mHSPC^69^, will be sufficient to uncover a subset of ICI-responsive patients missed in unstratified trials. Another possibility is that different therapeutic approaches may be appropriate in PCa, perhaps those that focus on mobilizing B cells or activate T cells by mechanisms that do not involve inhibition of PD-1-PD-L1 binding. One limitation of the current study in this respect is that our reliance on archival FFPE tissue, and the complex properties of pro-tumorigenic B-reg cells^70^, makes it difficult to determine whether the dense B cell infiltrate we have detected in PCa have only anti-tumor roles or whether they might also be immunosuppressive. Additional characterization of B cells in PCa is therefore warranted.

## METHODS

### Imaging and data generation

The extended versions of the marker names are provided in **Supplementary Table 1**, along with their associated antibodies and vendor information in **Supplementary Table 2**. All images were visually inspected, and the segmentation mask dataset was obtained using MCMICRO^44^, which generated the cell-feature table for each specimen. After multiple rounds of inspection, we excluded areas of poor quality from our analysis using in-house tools. The suitability of the markers for the analysis was also checked visually, with emphasis placed on properly stained markers of acceptable quality (Extended Data Fig. 1a). Similarly, the phenotyping used to determine cell identities was based on markers that had passed the previous quality control step (Extended Data Fig. 1b). We also used visual inspection to determine the marker intensity threshold above which a cell was labeled positive for that marker. For markers with overall poor quality but acceptable quality in the BIC areas (Ki67, TCF1, PD-1), we determined the positivity threshold only in these regions of interest. In all BICs where gating on the above markers was possible, we observed a subset of Ki67^+^ B cells that also scored as TCF1^+^. While it is possible that these represent a recently identified self-renewing population of B-1 cells^71^, we do not rule out that this may be due to quality issues in the segmentation of immune cells in densely packed ICs, leading to signal spillover from neighboring cells (Extended Data Fig. 3c). Therefore, we suggest that TCF1^+^ B cells may be artifacts resulting from poor segmentation.

### Special samples handling

**Extended Data Fig. 7 |:**
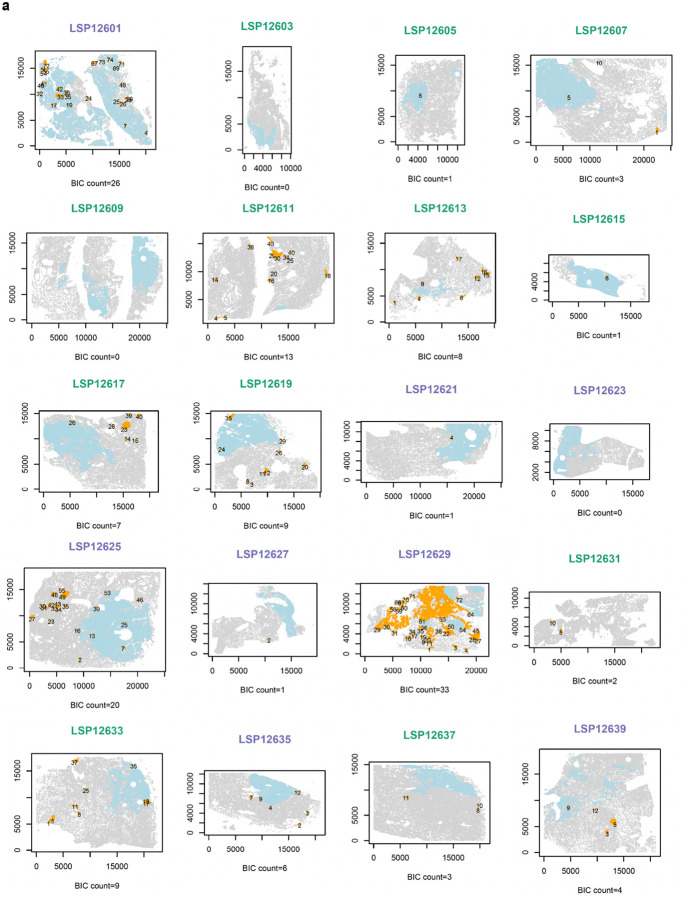

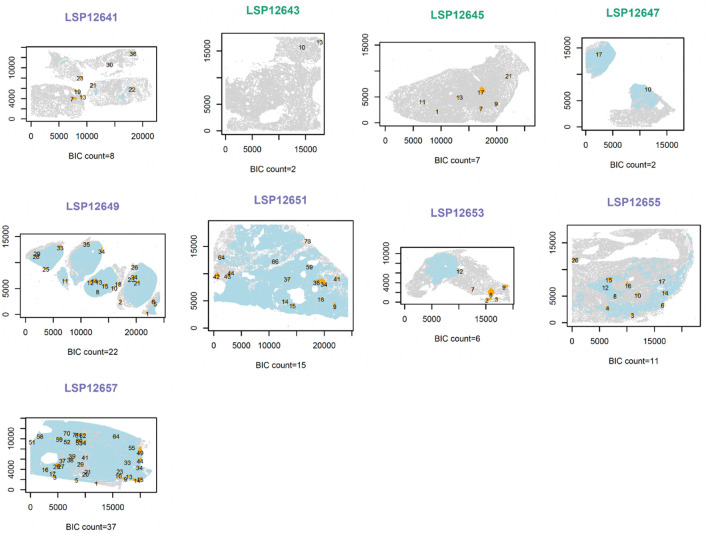
Tumor and BIC annotations across all samples. **a** Pseudo-images (cells are circles) of the whole-slide tissue annotated with the tumor areas (light blue) and numbered detected BICs in orange with their identifier. Titles stratified for HGG and LGG specimen with purple and green, respectively and the axes denote the coordinates in μm.

In the case of B cells, the considerably higher variance was largely driven by a single outlier case (LSP12629), which had nearly 10-fold more B cells than the next highest tumor. This tumor exhibited an extremely dense CD20^+^ B cell infiltrate winding in and out of the tumor compartment. Upon image review, these cells showed highly specific membranous staining, abundant CD19 staining, and very little off-target marker expression (e.g., CD3d), all supporting a bona fide B cell response rather than a technical artifact (Extended Data Fig. 7). Due to the extensive B cell proportion in this sample, we suspected that this patient had a concurrent or prior history of B-cell lymphoma. To avoid bias in our analysis, we excluded this sample from the infiltration analysis. Additionally, two detected BICs in this patient, each exceeding 10^5^ cells in size, were excluded from all BIC-related analyses. Three slides from LGG cases (LSP12631, LSP12643, LSP12645) did not have a tumor compartment visible on the image, although their grade assignments were based on whole-block review by pathologists. These samples were also excluded from analyses pertaining to the tumor, such as infiltration and proximity. In contrast, two LGG samples (LSP12603 and LSP12609) and one HGG sample (LSP12623) lacked B immune clusters and were therefore excluded from the proximity chart analysis.

### Comparison to colorectal cancer and infiltration

To gauge the absolute magnitude of immune infiltration, we calculated the median CD8^+^ and CD20^+^ tumor immune infiltration from a cohort of 74 colorectal cancers (CRC) previously imaged and analyzed using equivalent multiplexed IF technology and software. This cohort encompassed 65 patients with mismatch-repair proficient (pMMR) tumors and 9 patients with mismatch-repair deficient (dMMR) tumors^45^. Owing to deficiencies in nucleotide repair machinery, dMMR tumors have a significantly higher tumor mutational burden (TMB) and immune infiltrate compared with pMMR tumors and are primarily treated with first-line immunotherapy in the metastatic setting. The infiltration of B, CD4, and CD8 T cells was calculated by identifying all cells within areas annotated by expert pathologists as the tumor compartment and expanding a circular area around each cell with a 20 μm radius. Overlapping areas from adjacent cells were included only once. The number of specific lymphocytes in the annotated area was then divided by the total area obtained in the previous step to derive the infiltration per mm^2^.

### Statistical analysis

Multiple statistical tests were employed in this study, each selected based on the type of comparison and the most common assumptions about the data. Basic comparisons of central tendencies between two samples were performed using a *t*-test in cases with more than 25 data points per sample or when the normality assumption of the data distribution was not rejected by the Kolmogorov-Smirnov normality test; otherwise, the nonparametric Mann-Whitney U test was used. Multi-sample analyses were conducted with the nonparametric Kruskal-Wallis test. For assessing increasing trends in lymphocyte data, the Jonckheere-Terpstra trend test was applied. Infiltration values were compared with colorectal cancer counterpart values using the nonparametric Wilcoxon rank-sum test, while Levene’s test was used to compare variance between competing groups. The proportion z-test was used for comparing two proportion vectors, and for proportion vectors involving more than one subtype, dissimilarities were quantified using Jensen-Shannon divergence. For regression analyses, models were fitted with an intercept, and the overall goodness-of-fit was reported via the *F*-test (Fisher exact test) *P* value. In scenarios where the number of data points in one sample was more than twofold that of the other, the *R*^2^ value was also reported. The same handling of special samples (as discussed above) was applied to relevant regression analyses. In logarithmic regression analyses, samples with original zero values were excluded, as these were associated with suboptimal areas for gating specific markers. Box and violin plots, as well as regression fits, were generated based on Tukey’s outlier rule. UMAP projections were constructed using scaled marker intensities underlying the respective phenotypes, color-coded by the discrete assigned phenotypes, with parameters of 15 neighbors, a minimum distance of 0.08, and the Euclidean metric. The detailed mathematical derivation of ICAT, along with notes on the centered L-function (CLF), multitype L-function (MCLF), and derived summarization indices, are presented in Supplementary Data S1–2. All packages used in this study, along with their respective applications and native environments, are provided in **Supplementary Table 3**.

### Detection of the BIC and TIC

We used the K nearest neighbors (KNN) algorithm to detect B immune clusters (BICs). First, we identified clusters of cells where at least 50% of the 25-nearest neighbors were B cells. These were then expanded to include any neighboring immune cells (positive for CD11b, CD68, CD163, CD4, CD3d, CD8a, TCF1, FOXP3, PD-1, CD57, CD11c, GZB, CD15, HLA-DR, CD103, CD31, pTBK1, CD24, CD44, or CD206) if at least 20% of the 100 nearest neighbors of those immune cells were B cells. A minimum cluster eligibility cutoff of 300 cells was then applied. For T immune clusters (TICs), we used the DBSCAN algorithm, nucleated on T cells (positive for CD3d, CD4, and CD8), with a minimum cluster size cutoff of 100 cells and an expanding neighborhood radius of 50 μm to include proximate T cells.

### The spatial characterization with ICAT

To quantify the organization of ICs, we employed independent component analysis (ICA), a computational method that decomposes input variables into the most statistically independent components^47^. As a result, the transformed variables are uncorrelated and thus exhibit zero covariance in the off-diagonal elements of their covariance matrix, making the trace of the standard deviation matrix an indicator of spatial dispersion. Intuitively, when ICA is applied to the two-dimensional (x, y) spatial coordinates of cells, BICs with primarily circular morphology (sections from spherical TLSs) exhibit nearly perpendicular ICA component axes, in contrast to more oblique or acute axes for amorphous clusters (Fig. 4a). After normalization and applying a constant multiplier, we used the trace of the standard deviation matrix of the resulting new coordinates (termed ICAT) as a measure of the per-cell spatial standard deviation for each BIC (Supplementary Data S1). Supplementary Table 4 lists the TLS identifiers, ICAT values, pathologist annotations, and relevant marker expressions.

### The K-function and co-clustering

The positions of cells on the tissue can be modeled using a Poisson point process (PPP) if their spatial locations are simplified to single x-y coordinates. Ripley's K-function can then be used to quantify the spatial clustering or regularity tendencies of these points^72,73^. Under complete spatial randomness (CSR), the K-function is expected to expand exponentially with increasing radius, whereas Besag’s L-function (or centered L-function; CLF) shows a linear relationship with radius^74^. Consequently, dividing the CLF by the radius (*r*) results in a constant value under CSR (Fig. 5a; Extended Data Fig. 5a). Upward deviations from this constant indicate clustering intensity, while values below it suggest regularity^75^.

For our cohort-based analysis, we computed the CLF up to a 1.5 mm radius, using increments of 30 μm, separately for B and T cells. Tissue-wise curves were generated by averaging the estimated divergences of the CLF from CSR at each radius interval, across four bootstrap iterations of squared 9 mm^2^ tiles from all tissues. The bootstrapping step was included to mitigate bias from suboptimal tissue bracketing. Using identical parameters, the multitype L-function (MCLF) was estimated by counting the number of T cells within the radius of each B cell (Supplementary Data S2). The 9 mm^2^ tile size was reverse-optimized by examining the behavior of the CLF and MCLF across a range of radii, ensuring capture of all interaction dynamics and a confident downward trend toward CSR within this window. For exemplary cases, we computed the CLF and MCLF over a 500 μm radius from each cell, using 10 μm increments (Extended Data Fig. 5a). To derive meaningful insights from the resulting CLF curves, we characterized them along two orthogonal dimensions: clustering intensity (CI), defined as the integral of the curve in its positive r domain; and R50, the maximum radius at which the curve accounts for 50% of the positive integral area. We also defined the effective clustering radius (ECR) as the maximum radius above which the curve enters the 95% confidence interval around CSR; due to the right-skewness of the L-function, this was conservatively estimated by doubling R50. Analogous metrics—co-clustering intensity (CCI) and estimated co-clustering radius (ECCR)—were derived using the MCLF instead of the CLF (Supplementary Data S2).

## Extended Data

## Supplementary Material

1

## Data Availability

All CyCIF images (across all channels) and digitized H&E images will be deposited to AWS servers, with an accessible link provided upon acceptance of this manuscript. The segmented data (feature tables), higherlevel processed data, and additional resources required to reproduce the analyses will be deposited in Zenodo and assigned a permanent DOI. During peer review, these datasets will be made available to editors and reviewers upon request.

## Code availability

All code necessary to reproduce the results will be released on GitHub following acceptance of this manuscript. For the purposes of editorial assessment and peer review, the code will be made available upon request.
