# Supplementary material for: Spatially organized lymphocytic microenvironments in high grade primary prostate tumors
Source: bioRxiv. 2025 Sep 21:2025.09.21.677465. Preprint. [Version 1] doi: 10.1101/2025.09.21.677465 (PMC12458951; doi:10.1101/2025.09.21.677465)
Supplement: 1 [file NIHPP2025.09.21.677465V1-supplement-1.pdf]

bioRxiv preprint doi: https://doi.org/10.1101/2025.09.21.677465; this version posted September 21, 2025. The copyright holder for this preprint (which was not certified by peer review) is the author/funder, who has granted bioRxiv a license to display the preprint in perpetuity. It is made available under aCC-BY 4.0 International license.

Supplementary Data

S1. Independent component analysis of the immune clusters

The goal is to find the aggregated standard deviation of the points in the x-y space per cells in a given immune cluster (IC) by prioritizing the canonical shapes. Note that we can use the Independent Component Analysis (ICA) matrix decomposition for cells in IC $\mathbf{X}$ as

$$\mathbf{X} = \begin{bmatrix} x_1 & y_1 \\ x_2 & y_2 \\ \vdots & \vdots \\ x_n & y_n \end{bmatrix}_{n\times 2} \quad ICA \quad \longrightarrow \quad \widehat{\mathbf{X}} = \mathbf{S}\,\mathbf{A}^{\mathsf{T}} + \mathbf{1}\mu^{\top}, \tag{1}$$

where $\mathbf{S} = [\,\mathbf{s}_1 \;\; \mathbf{s}_2\,]_{n\times 2}$ are the **independent components**, $\mathbf{A} \in \mathbb{R}^{2\times 2}$ is the **mixing matrix**, $\mathbf{1} \in \mathbb{R}^n$ is the all-ones vector, and $\mu = \begin{bmatrix} \mu_x \\ \mu_y \end{bmatrix} = \frac{1}{n}\sum_{i=1}^{n} \begin{bmatrix} x_i \\ y_i \end{bmatrix}$.

The $\widehat{\mathbf{X}}$ is the 'best' independent representation of the X in the same unit space

$$\widehat{\Sigma} = \begin{bmatrix} \widehat{\sigma}_x^2 & \widehat{\sigma}_{xy} \\ \widehat{\sigma}_{xy} & \widehat{\sigma}_y^2 \end{bmatrix} \tag{2}$$

as the covariance matrix with trace and determinant of

$$t = tr(\widehat{\Sigma}) = \widehat{\sigma}_x^2 + \widehat{\sigma}_y^2, \quad \Delta = det(\widehat{\Sigma}) = \widehat{\sigma}_x^2\widehat{\sigma}_y^2 - \widehat{\sigma}_{xy}^2. \tag{3}$$

Now the standard deviation matrix is given by

$$\widehat{\epsilon} = \widehat{\Sigma}^{1/2} = \frac{\widehat{\Sigma} + \delta\mathbf{I}}{\sqrt{t + 2\delta}}, \tag{4}$$

where $\delta = \sqrt{\Delta}$, $\mathbf{I}$ is the identity matrix, and $\sqrt{t + 2\delta}$ is

$$(\widehat{\sigma}_x^2 + \widehat{\sigma}_x^2 + 2\sqrt{\widehat{\sigma}_x^2\widehat{\sigma}_y^2 - \widehat{\sigma}_{xy}^2})^{1/2} \approx (\widehat{\sigma}_x^2 + \widehat{\sigma}_y^2 + 2\widehat{\sigma}_x\widehat{\sigma}_y)^{1/2} = T \tag{5}$$

which is so as under statistical independence assumption of $\widehat{\mathbf{x}} \perp \widehat{\mathbf{y}}$, we have $\widehat{\sigma}_{xy} = \widehat{\sigma}_{yx} \approx 0$ tacitly penalizing cases where correlation between $\mathbf{x}'$ and $\mathbf{y}'$

bioRxiv preprint doi: https://doi.org/10.1101/2025.09.21.677465; this version posted September 21, 2025. The copyright holder for this preprint (which was not certified by peer review) is the author/funder, who has granted bioRxiv a license to display the preprint in perpetuity. It is made available under aCC-BY 4.0 International license.

still persist underlying point configurations with aberrant non-collinear shapes that independent components are not perfectly achievable. This leads to $T = \sqrt{(\widehat{\sigma}_x + \widehat{\sigma}_y)^2} = \widehat{\sigma}_x + \widehat{\sigma}_y$ which is exactly the trace of the standard deviation matrix $tr(\widehat{\Sigma}^{1/2})$.

So the per-cell projected (percentage if $\alpha = 100$) and normalized total standard deviation of a cluster based on the underlying independent coordinates is

$$ICAT = \alpha T/n. \tag{6}$$

S2. Ripley $K-$, $L-$functions and derived clustering indices

Let

$$\Phi_B = \{x_1^{(B)}, \dots, x_{n_B}^{(B)}\}, \qquad \Phi_T = \{x_1^{(T)}, \dots, x_{n_T}^{(T)}\} \subset W \subset \mathbb{R}^2,$$

with $|W|$ the tissue area and $\lambda_B = n_B/|W|$, $\lambda_T = n_T/|W|$ the global intensities.

Theoretical $K-$functions. For a single type $i \in \{B, T\}$

$$K_{ii}(r) = \frac{1}{\lambda_i}\,\mathbb{E}\Big[N\{x_j^{(i)} \neq o : \|x_j^{(i)} - o\| \leq r\} \;\Big|\; o \in \Phi_i\Big], \tag{7}$$

and for cross-type interaction $(i \neq j)$

$$K_{ij}(r) = \frac{1}{\lambda_j}\,\mathbb{E}\Big[N\{x_k^{(j)} : \|x_k^{(j)} - o\| \leq r\} \;\Big|\; o \in \Phi_i\Big]. \tag{8}$$

Edge-corrected estimator.

$$\widehat{K}_{ij}(r) = \frac{|W|}{n_i\, n_j} \sum_{x\in\Phi_i} \sum_{y\in\Phi_j\setminus\{x\}} \frac{\mathbf{1}\big(\|x-y\| \leq r\big)}{e_W(x,y)}, \tag{9}$$

where $\mathbf{1}(\|x-y\| \leq r) = 1$ when the inter-cell distance does not exceed r, and $e_W(x, y)$ is Ripley's isotropic edge weight.

Variance-stabilized and centralized $L-$function

$$\widehat{L}_{ij}(r) = \sqrt{\widehat{K}_{ij}(r)/\pi}, \qquad \Delta\widehat{L}_{ij}(r) = \widehat{L}_{ij}(r) - r, \tag{10}$$

so that $\Delta\widehat{L}_{ij}(r) > 0$ signals attraction, $= 0$ CSR, and < 0 inhibition.

bioRxiv preprint doi: https://doi.org/10.1101/2025.09.21.677465; this version posted September 21, 2025. The copyright holder for this preprint (which was not certified by peer review) is the author/funder, who has granted bioRxiv a license to display the preprint in perpetuity. It is made available under aCC-BY 4.0 International license.

Positive envelope. Define $g_{ij}(r) = \max\{0, \Delta\widehat{L}_{ij}(r)\}$ and denote by $R_{ij}^{+} = \sup\{r : g_{ij}(r) > 0\}$ the largest radius at which any positive deviation persists.

1. **Clustering intensity**

$$\mathrm{CI}_{ij} = \frac{1}{R_{ij}^{+}} \int_0^{R_{ij}^{+}} g_{ij}(r)\, dr, \tag{11}$$

   i.e. the mean magnitude of attraction across the range on which it is present.

2. **Half-mass radius**

$$\mathrm{R50}_{ij} = \inf\Big\{r : \int_0^{r} g_{ij}(t)\, dt \geq \tfrac{1}{2} \int_0^{R_{ij}^{+}} g_{ij}(t)\, dt\Big\}, \tag{12}$$

   the radius enclosing first-half of the *total positive clustering mass*.

3. **Effective clustering radius**

$$\mathrm{ECR}_{ij} = 2\,\mathrm{R50}_{ij} \pm \epsilon, \tag{13}$$

   providing an operational outer limit of attraction that remains slightly inside the point where $\Delta\widehat{L}_{ij}(r)$ first returns to the CSR baseline, where ϵ is half of the binning used for r toward estimation of the $K-$function.

Together, CI_{ij}, $\mathrm{R50}_{ij}$, and ECR_{ij} compress the full centred L–curves into three interpretable numbers describing, respectively, the average strength, the core scale, and the effective spatial extent of B–, T–, and B–T cells interactions.
